# The mitochondrial genome of the steppe carpenter moth (*Paracossulus thrips* Hübner, 1818): Structural analysis and phylogenetic implications

**DOI:** 10.1038/s41598-025-93646-6

**Published:** 2025-03-11

**Authors:** Sándor Jordán, Levente Laczkó, Szilárd Póliska, Tamás Korompai, Gábor Sramkó

**Affiliations:** 1 Evolutionary Genomics Research Group, Department of Botany, Faculty of Science and Technology, University of Debrecen, Egyetem tér 1, Debrecen, 4032 Hungary; 2 HUN-REN–UD Conservation Biology Research Group, Egyetem tér 1, Debrecen, 4032 Hungary; 3 Juhász-Nagy Pál Doctoral School, University of Debrecen, Egyetem tér 1, Debrecen, 4032 Hungary; 4 One Health Institute, University of Debrecen, Nagyerdei körút 98, Debrecen, 4032 Hungary; 5 Department of Biochemistry and Molecular Biology, Faculty of Medicine, University of Debrecen, Egyetem tér 1, Debrecen, 4032 Hungary; 6 Bükk National Park Directorate, Sánc u. 6, Eger, 3304 Hungary

**Keywords:** Evolutionary genetics, Mitochondrial genome, Phylogenetics, Entomology, Conservation biology

## Abstract

*Paracossulus thrips* (Lepidoptera: Cossoidea) is a rare micromoth species native to the Eurasian steppe that occurs in fragmented populations across its distribution area. In Europe, it persisted only in a few isolated populations, which warranted protection by the EU’s Habitats Directive. We assembled the first complete mitochondrial genomes of two individuals of *P. thrips* using shotgun whole-genome sequencing data. The assembled mitogenomes were complete and circular; they contained 13 protein-coding genes, 22 tRNA genes, and two rRNA genes. The A + T-rich control region (CR) was identified between the 12 S rRNA and tRNA-*Met* (CAU) regions. We performed phylogenetic tree reconstruction focusing on the Cossoidea superfamily within the Lepidoptera order by incorporating the new mitochondrial genome assemblies presented in this study. Using available mitogenomes of the superfamily, the mitochondrial phylogeny placed *P. thrips* within the Cossinae subfamily as a sister to the only other species with an assembled mitogenome. These assemblies may provide valuable genetic resources for further large-scale phylogenetic studies of the Cossoidea superfamily, a poorly studied group of the Lepidoptera order. This work could also support the long-term conservation management of this unique species by providing resources for conservation genetic research.

**Supplementary Information:**

The online version contains supplementary material available at 10.1038/s41598-025-93646-6.

## Introduction

The evolutionary history of Lepidoptera, one of the most emblematic insect orders, has advanced exponentially as studies employing different methodologies have revealed various aspects of the phylogenetics of this order. Notably, those based on the analysis of morphological characters^1^ and molecular genetic data^2–5^, including the analysis of whole mitochondrial genomes^6–9^, greatly aided the in-depth understanding of the evolution of the order Lepidoptera. Despite extensive studies, many of which have broad taxonomic representation, the evolutionary history within certain lepidopteran superfamilies remains unclear. This uncertainty is also noticeable in the basal or so-called non-Obtectomera superfamilies of the Apoditrysia crown-clade. The phylogenetic relationship of the 14–15 superfamilies considered to belong to this group^10,11^ remained poorly understood and often described as unresolved and treated as polytomous^12^. Phylogenetic studies on these superfamilies often yield contradictory results. Studies applying different methodologies, such as morphological data combined with a few genes^3^ or integrating mitochondrial and nuclear regions^13^ resulted in conflicting conclusions. Genome-scale molecular data is a powerful resource for reconstructing and understanding the evolutionary processes of challenging groups such as the basal apoditrysian superfamilies. However, until recently, these lineages lacked sequenced genomes^14^, and the absence of molecular genetic data has created substantial uncertainties surrounding the basal Apoditrysia superfamilies, despite the economic, taxonomic, and conservation importance of their constituent species. While ongoing genome sequencing efforts have begun to address this gap, the availability of genome-scale molecular data remains strikingly limited in some basal apoditrysian superfamilies.

Cossoidea is one of the well-recognized superfamilies within the non-obtectomeran Apoditrysia clade, including *Paracossulus*, a monotypic genus represented by *Paracossulus thrips *(Hübner, 1818) (Cossoidea: Cossidae: Cossinae)^15^. This understudied species is a rare component of the Eurasian fauna with high conservation importance. Its distribution area spans longitudinally from southwestern Siberia (Altai region) to Central Europe (Hungary) and extends southward to Iran and Turkey^16,17^. At the westernmost edge of its range, the species persists in fragmented populations in Bulgaria^18–20^, Hungary^21^, Romania^22,23^, Serbia^20^, and with a single historical record from southeastern Poland^24^. In the European Union, it is protected by the EU Council Directive (NATURA2000 code: 4028) but is not included in the IUCN Red List. The species is classified as critically endangered in Bulgaria^18^, endangered in Hungary^25^, and vulnerable in Romania^23^. The species is threatened primarily by habitat loss due to urbanization, agricultural activities, or other infrastructural developments^19,20,22^.

The knowledge of *P. thrips *was severely limited for a long time, with only some recent studies^20,22,26^ providing new insights, including indications of its ecological demands. The first study^22^ employing molecular markers to study this species presented the first and, so far, the only available molecular genetic data of the species: partial *cytochrome c oxidase I* (COI or *cox1*) sequences of four specimens. This study confirmed the phylogenetic position of *P. thrips* within the Cossoidea superfamily, consistent with the earlier morphology-based taxonomic classifications, and confirmed the monotypic nature of the genus. However, to gain a deeper understanding of the broader phylogenetic relationships and to provide effective molecular tools for conservation management for this species, generating genome-level data is essential.

In this study, we aimed to sequence and assemble the mitochondrial genome of two *P. thrips* individuals from the same population. We then characterized these genomes to provide valuable resources for evolutionary and conservation research of the Cossoidea superfamily—a largely understudied group within the Lepidoptera order.

## Results

### Whole-genome sequencing

We obtained whole-genome sequencing datasets from two individuals on an MGI DNBSEQ-G400RS platform. Whole genome sequencing yielded 150 bp long paired-end reads, totaling 43.9 gigabasepair (Gbp) (292,983,070 reads) for the male individual (Sample ID: CAT07) and 40.7 Gbp (271,272,886 reads) output for the female individual (Sample ID: CAT08). Given that this dataset consists solely of short reads, it is not suitable for *de novo* assembly of a high-quality nuclear genome. Therefore, in this paper, we focused only on the organellar reads derived from these datasets.

Of the raw reads, 1,234,704 (0.185 Gbp, 0.42%) from the CAT07 sample and 949,376 (0.142 Gbp, 0.35%) from the CAT08 sample were aligned to the reference mitochondrial genome of *Eogystia hippophaecolus*. The mean depth of coverage of the aligned reads was 7,451 for CAT07 and 6,103 for CAT08.

### General characteristics of the assembled mitogenomes

**Fig. 1:**
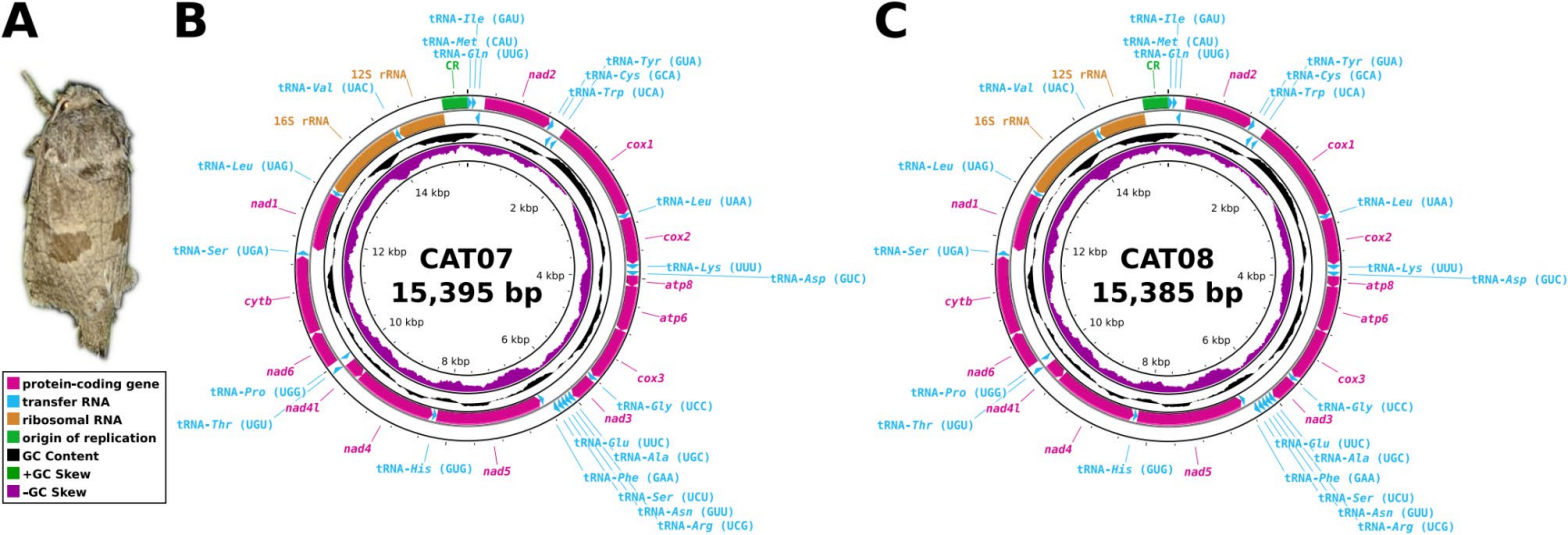
The *Paracossulus thrips* and the circular maps of the two mitochondrial genome assemblies. **A**: A female specimen of *P. thrips* (Photo: Sándor Jordán); **B**: Circular map of the mitochondrial genome of the male (CAT07) specimen; **C**: Circular map of the mitochondrial genome of the female (CAT08) specimen. The bars surrounding the maps’ backbones represent the genetic regions identified within the mitogenomes. The orientation of the arrowheads at the ends of the bars indicates the strand on which each gene is encoded and the direction of translation for the protein-coding genes.

The assembled mitochondrial genomes were complete and circular with lengths of 15,395 bp for the male specimen (CAT07) and 15,385 bp for the female specimen (CAT08). Both mitogenomes contain 13 protein-coding genes (PCGs), 22 tRNA coding regions, two rRNA coding regions, and an A + T-rich non-coding control region (CR), and 19 shorter (1–62 bp) intergenic non-coding spacers (Fig. 1; Table 1). The majority of PCGs and tRNA genes, along with the CR, are located on the heavy strand, whereas *nad1*, *nad4*, *nad4l*, and *nad5* PCGs, and the tRNA-*Cys* (GCA), tRNA-*Gln* (UUG), tRNA-*His* (GUG), tRNA-*Leu* (UAG), tRNA-*Phe* (GAA), tRNA-*Pro* (UGG), tRNA-*Tyr* (GUA), and tRNA-*Val* (UAC) tRNA genes, as well as both rRNA genes, are located on the light strand.

**Table 1:** Annotation of the two mitochondrial genome assemblies of *Paracossulus thrips*.

Gene	Location		Strand	Length (bp)		Overlaps (bp)		Codons	
	CAT07	CAT08		CAT07	CAT08	CAT07	CAT08	Start	Start
tRNA-*Met* (CAU)	1–66	1–66	+	66	66	0	0		
tRNA-*Ile* (GAU)	67–130	67–130	+	64	64	−3	−3		
tRNA-*Gln* (UUG)	128–196	128–196	-	69	69	62	62		
*nad2*	259–1,272	259–1,272	+	1,014	1,014	−2	−2	ATT	TAA
tRNA-*Trp* (UCA)	1,271–1,340	1,271–1,340	+	70	70	−8	−8		
tRNA-*Cys* (GCA)	1,333–1,401	1,333–1,401	-	69	69	35	37		
tRNA-*Tyr* (GUA)	1,437–1,501	1,439–1,503	-	65	65	3	3		
*cox1*	1,505–3,040	1,507–3,042	+	1,536	1,536	−5	−5	CGA	TAA
tRNA-*Leu* (UAA)	3,036–3,105	3,038–3,107	+	70	70	0	0		
*cox2*	3,106–3,787	3,108–3,789	+	682	682	3	3	ATG	T
tRNA-*Lys* (UUU)	3,791–3,862	3,793–3,864	+	72	72	30	30		
tRNA-*Asp* (GUC)	3,893–3,959	3,895–3,961	+	67	67	0	0		
*atp8*	3,960–4,124	3,962–4,126	+	165	165	−7	−7	ATT	TAA
*atp6*	4,118–4,795	4,120–4,797	+	678	678	−1	−1	ATG	TAA
*cox3*	4,795–5,583	4,797–5,585	+	789	789	2	2	ATG	TAA
tRNA-*Gly* (UCC)	5,586–5,652	5,588–5,654	+	67	67	0	0		
*nad3*	5,653–6,006	5,655–6,008	+	354	354	5	5	ATT	TAA
tRNA-*Ala* (UGC)	6,012–6,079	6,014–6,081	+	68	68	−1	−1		
tRNA-*Arg* (UCG)	6,079–6,142	6,081–6,144	+	64	64	0	0		
tRNA-*Asn* (GUU)	6,143–6,207	6,145–6,209	+	65	65	1	1		
tRNA-*Ser* (UCU)	6,209–6,274	6,211–6,276	+	66	66	7	7		
tRNA-*Glu* (UUC)	6,282–6,350	6,284–6,352	+	69	69	45	35		
tRNA-*Phe* (GAA)	6,396–6,462	6,388–6,454	-	67	67	1	1		
*nad5*	6,464–8,200	6,456–8,192	-	1,737	1,737	0	0	ATT	TAA
tRNA-*His* (GUG)	8,201–8,267	8,193–8,259	-	67	67	0	0		
*nad4*	8,268–9,606	8,260–9,598	-	1,339	1,339	1	1	ATG	T
*nad4l*	9,608–9,895	9,600–9,887	-	288	288	9	9	ATG	TAA
tRNA-*Thr* (UGU)	9,905–9,969	9,897–9,961	+	65	65	0	0		
tRNA-*Pro* (UGG)	9,970–10,035	9,962–10,027	-	66	66	2	2		
*nad6*	10,038–10,568	10,030–10,560	+	531	531	11	11	ATT	TAA
*cytb*	10,580–11,728	10,572–11,720	+	1,149	1,149	14	14	ATG	TAA
tRNA-*Ser* (UGA)	11,743–11,809	11,735–11,801	+	67	67	17	17		
*nad1*	11,827–12,768	11,819–12,760	-	942	942	1	1	ATG	TAA
tRNA-*Leu* (UAG)	12,770–12,838	12,762–12,830	-	69	69	0	0		
16 S rRNA	12,839–14,174	12,831–14,164	-	1,336	1,334	1	1		
tRNA-*Val* (UAC)	14,176–14,244	14,166–14,234	-	69	69	0	0		
12 S rRNA	14,245–15,020	14,235–15,010	-	776	776	0	0		
CR	15,021–15,395	15,011–15,385	+	375	375	-	-		

The “Location” column indicates position of each gene within the assembly, while the “Strand” column shows the orientation of the gene: genes encoded on the heavy strand are marked with a “+”, and genes encoded on the light strand are marked with a “-”. The “Overlaps (bp)” column refers to overlapping or intergenic nucleotides, where a value of 0 indicates no overlap or intergenic between two genes, a positive value indicates non-coding intergenic nucleotide(s), while a negative value indicates overlapping nucleotide(s) with the following region. The “CR” refers to the “control region”, commonly referred to as the A-T-rich non-coding region.

A total of 11 mutations were identified between the two assemblies (Table S2). These included six single nucleotide polymorphisms (SNPs), two insertion/deletion (indel) mutations, and three microsatellites. Among the SNPs, five were transitions and one was a transversion. Of these, one occurred in the A + T-rich non-coding region, one in a tRNA gene, and four in the PCGs. One of the mutations in the PCGs was silent, while the remaining three led to changes in the amino acid sequences of the encoded proteins. The microsatellites were exclusively located in the intergenic regions (Table S2).

The total base composition of the assembled mitogenomes was nearly identical between samples: A: 39.9%, C: 14.5%, G: 7.8%, and T: 37.8% in the CAT07, and T: 37.7% in the CAT08 sample. Based on the base content, the AT-skewness was slightly positive (0.03), while the GC-skewness had a negative value (−0.30) in both mitochondrial genome assemblies (Table S3).

### Protein-coding genes

The lengths of the PCGs ranged between 165 bp (*atp8*) and 1,737 bp (*nad5*). The majority of the PCGs initiated with ATN codons. Among these, the ATG start codon was observed in seven genes (*atp6*, *cox2*, *cox3*, *cytb*, *nad1*, *nad4*, *nad4l*), while five genes (*atp8*, *nad2*, *nad3*, *nad5*, *nad6*) were initiated with ATT codon (Table 1). As an exception, CGA start codon was identified in the *cox1* gene (Table 1) which is commonly observed in Lepidoptera species^27,28^. Most PCGs terminated with the TAA stop codon, however, an abbreviated stop codon (T) was detected in the *cox2* and *nad4* genes. Four point mutations were identified across four distinct PCGs (*cox1*, *cox3*, *nad1*, *nad4l*) between the two mitogenomes (Table S2). These mutations, comprising three transitions and one transversion, all resulted in non-synonymous codon changes, regardless of their position within the codons. The transversion in the *nad4l* gene affected the third codon position replacing the ATA (Met) codon with ATT (Ile). The transitions altered the first codon positions leading to the replacement of GCC(Ala) for ACC (Thr) in *cox1*, AGA (Ser) for GGA (Gly) in *cox3*, and CCT (Pro) for TCT (Ser) in *nad1* (Table S2).

All PCGs were characterized by a generally higher proportion of A and T bases with their content ranging from 68.1 to 88.5% (average = 76.7%). The AT-skewness had an average negative value (−0.13), ranging from − 0.29 to −0.01, indicating a higher abundance of T over A in the protein-coding genes. GC-skewness varied between − 0.68 and 0.53 (average = −0.09), reflecting a generally higher prevalence of C over G in the PCGs, except for a few genes (*nad*1, *nad*4, *nad*4*l*, and *nad*5) where G was more abundant (Table S3).

Analysis of amino acid frequency (Fig. S1) and relative synonymous codon usage (RSCU) (Fig. S2) showed that leucine (L), isoleucine (I), phenylalanine (F), and serine (S) were the four most frequently encoded amino acids, while cysteine (C) was the least common. Analysis of the RSCU also revealed a higher occurrence of codons with A or T, a commonly observed pattern in Lepidoptera mitogenomes^29^.

### Transfer RNA and ribosomal RNA genes

The lengths of the tRNA genes ranged from 64 bp to 72 bp. Based on the predicted secondary structures (Fig. S3), most tRNA genes exhibited a cloverleaf structure. An exception was observed in tRNA-*Ser*(UCU), which lacked the dihydrouridine arm similar to several other arthropod species^7,9,28,30–32^.

The 12 S rRNA gene was 776 bp in both assemblies. In contrast, two length polymorphisms were detected in the 16 S rRNA gene (Table S2), with lengths of 1,336 bp in the CAT07 and 1,334 bp in the CAT08 sample.

Consistent with the higher A and T base content in the mitogenomes, the tRNA and rRNA genes also exhibited higher A + T levels, ranging from 72.7 to 92.8% in the tRNA genes and 82.0–85.2% in the rRNA genes. The AT-skewness values ranged from − 0.10 to 0.15 (average = 0.02) in the tRNA genes, and from − 0.04 to 0.03 in the rRNA genes, indicating an almost balanced ratio of A and T bases in these regions. The values of GC-skewness for the tRNA genes varied between − 0.33 and 0.50 (average = 0.15). In contrast, the rRNA genes had consistently positive and closely similar GC-skewness values ranging from 0.36 to 0.44. This suggests a higher proportion of G bases compared to C bases in the ribosomal RNA-coding genes (Table S3).

### Non-coding and overlapping regions

Several non-coding regions were identified in the assembled mitogenomes. The longest of these is a 375 bp non-coding region located between the 12 S rRNA and tRNA-*Met* (CAU) genes (Table 1), often referred to as the control region. This region exhibited a characteristically high A + T content in both assemblies, at 93.3% in CAT07 and 93.6% in CAT08. The AT-skewness value was slightly negative (−0.06), while the GC-skewness was distinctly negative with an average of −0.39. These values indicate a nearly balanced ratio of A and T bases, and a higher proportion of C bases compared to the G bases (Table S3). This region contains several conserved motifs typically found in Lepidoptera mitogenomes. Among these, the ‘ATAGA’ motif and the subsequent poly-T run ((T)_18_) are located upstream of the 12 S rRNA gene and are associated with the initiation of mitogenome replication^33^. Additionally, several A_n_ (*n* = 2–8) and T_n_ (*n* = 2–5) polynucleotide runs were identified throughout this region, including a shorter poly-A motif (4 bp long) at the end of this region, towards the tRNA-*Met*(CAU) gene, which is another frequently occurring feature in lepidopteran mitogenomes^28^.

The shorter intergenic spacers often consist of only a single base pair, while the longer ones may include repetitive structures (Table S2). The 17 bp long spacer found between the tRNA-*Ser* (UGA) and *nad1*genes contains the ‘ATACTAA’ motif, which is conserved among Lepidoptera species and associated with transcription termination^27,34^.

Shorter overlaps (1–8 bp) were identified between several genes (Table 1). Among these, the ‘ATGATAA’ sequence found in the overlap between the *atp8* and *atp6*genes is conserved across the majority of the Lepidoptera order^28^.

### Phylogenetic reconstruction

**Fig. 2:**
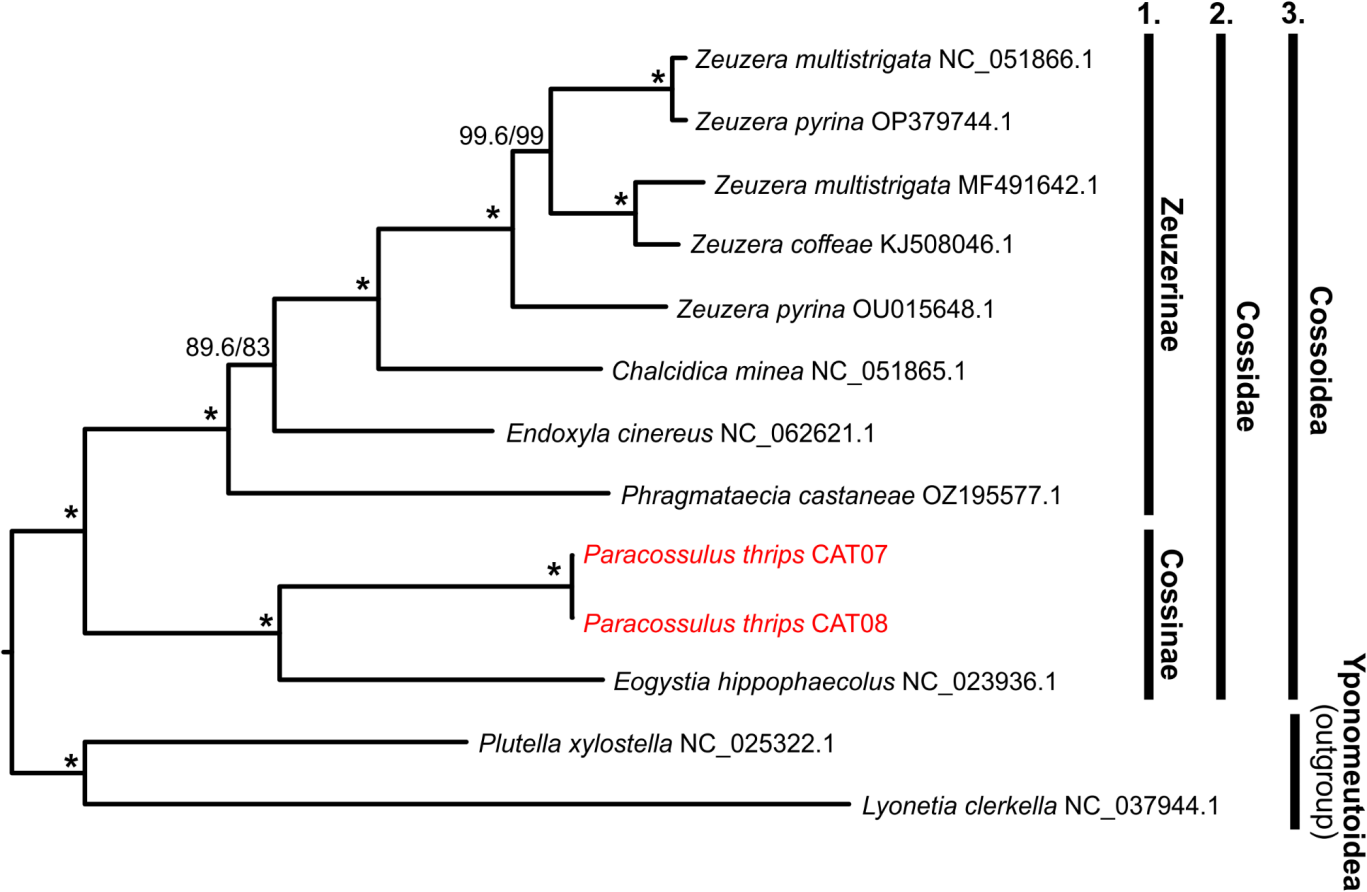
The reconstructed phylogenetic tree of Cossoidea. The present tree was reconstructed using the ML approach based on the merged nucleotide sequences of 13 mitochondrial PCGs with nucleotide substitution models. The phylogenetic tree shown here was reconstructed with all genes treated as distinct partitions. Branch support values represent SH-like approximate likelihood ratio test (SH-aLRT) and ultrafast bootstrap (UFboot) with 10,000 replicates. Branches with full statistical support (100/100) are marked with an asterisk (*). Bars on the right-hand side denote classification at the (1) subfamily, (2) family, and (3) superfamily level. Results from alternative partitioning schemes or substitution models are provided in Supplementary Fig. S4 A–C.

We conducted phylogenetic reconstructions using mitochondrial protein-coding genes to gain insight into the evolutionary relationships of the Cossoidea superfamily and to determine the phylogenomic position of *Paracossulus thrips* based on the currently available mitogenomes. The phylogenetic analyses yielded a consistent topology across all partitioning schemes (see Materials and Methods) and substitution models applied: all phylogenetic relationships were fully supported, excluding a single node at the basal position of Zeuzerinae (see below). Support for this basal node varied across analyses but remained low (Fig. S4). Since our partitioning scheme I) yielded the highest support for this node and was used for our primary result (Fig. 2). The reconstructed trees demonstrated high or full statistical support for nearly all branches (Fig. 2, Fig. S4). The ingroup (Cossoidea: Cossidae) formed a fully supported monophyletic unit. The diversification of two major clades was revealed within the ingroup corresponding to the Cossinae and Zeuzerinae subfamilies of the Cossidae family^2,15^. Our *Paracossulus thrips* specimens were placed as sisters to the *Eogystia hippophaecolus* on branches with full statistical support. Notably, these two species are the only representatives of the Cossinae subfamily, which formed a monophyletic group with full statistical support in all analyses (Fig. 2).

The remaining ingroup species formed another fully supported clade corresponding to the Zeuzerinae subfamily. However, the basal placement of *Phragmataecia castaneae* within Zeuzerinae remains equivocal (Fig. 2, Fig. S4). Despite the uncertain basal relationships within Zeuzerinae, subsequent nodes were strongly supported. First, *Chalcidica minea* was found on a fully supported branch, followed by all *Zeuzera* species forming the crown clade of the Zeuzerinae subfamily. However, our results revealed taxonomic inconsistencies within the *Zeuzera* samples. Specifically, the two *Z. multistrigata* samples and the two *Z. pyrina* samples were not recovered as sister groups (Fig. 2, Fig. S4). It is important to note that the *Z. coffeae* sample (KJ508046.1), which was positioned next to one of the *Z. multistrigata* specimens in our analyses, had only a fragment of the mitochondrial genome, consisting of only five genes. This resulted in missing data for most genes in this sample. Despite this shortcoming, all branches within the *Zeuzera* genus were strongly supported. The observed polyphyly within *Zeuzera* makes it unlikely that missing data in the *Z. coffeae* sample caused the polyphyletic placement of other species’ samples. An alternative explanation could be the misidentification of some samples that provided the mitogenomes involved in these analyses. Still, hybridization between closely related species or incomplete lineage sorting could also be invoked. However, tracing the reasons behind the taxonomic inconsistency found between our *Zeuzera* samples is beyond the scope of this study.

## Discussion

*Paracossulus thrips*is a rare and understudied moth species of the Eurasian steppe fauna. The accumulation of molecular data is highly desired for the conservation management of endangered species, underscoring the importance of genomic resources in this context^35^.

Herein, we present the first complete mitochondrial genomes of *P. thrips*, making it the second species within the Cossinae subfamily with a fully characterized mitogenome sequence. Given that this species belongs to the relatively understudied Cossoidea superfamily, these assemblies represent a valuable genetic resource for further research on this taxon.

The mitogenomes contained 13 protein-coding genes, 22 tRNA genes, two rRNA genes, a long A + T-rich non-coding region, and several short intergenic spacers ranging from 1 to 62 bp. The gene order follows the typical organizational pattern observed in most Lepidoptera mitogenomes^28^. The A-T-rich non-coding region is located between the 12 S rRNA and tRNA-*Met* (CAU) genes, and the arrangement of tRNA-*Met* (CAU), tRNA-*Ile* (GAU), tRNA-*Gln* (UUG) genes (Fig. 1; Table 1) are consistent with features shared by Lepidoptera mitochondrial genomes^28,31^. Furthermore, numerous conserved nucleotide motifs, characteristic of Lepidoptera mitogenomes, were also identified in the assembled mitogenomes.

Our phylogenetic reconstruction, despite limitations due to poor taxonomic representation because of the scarcity of available mitochondrial genomes for the Cossoidea superfamily, still allowed to draw informative taxonomic conclusions. The additional mitochondrial genomes included in the analyses were all from members of the Cossidae family as other Cossoidea families lacked mitochondrial genome representation in databases at the time of this study. Our results showed taxonomic consistency, with two major clades corresponding to the Cossinae and Zeuzerinae subfamilies. The examined *Paracossulus thrips* samples were placed sister to *Eogystia hippophaecolus* forming a monophyletic group representing the Cossinae subfamily. The close phylogenetic relationship between *P. thrips* and *E. hippophaecolus*is concordant with previous results^22^ based on the barcoding region of the *cox1* gene.

The complete mitochondrial genome sequences of *Paracossulus thrips*provide a valuable genomic resource for the overlooked and underrepresented group Cossinea subfamily within the Lepidoptera. In addition, these mitogenomes can be crucial resources for developing species-specific molecular markers that can be used for conservation management and biodiversity monitoring. Such markers enable non-invasive detection of the species’ persistence and distribution through environmental sample and biological remnant barcoding, facilitating taxonomic identification with basic laboratory equipment and skills^36,37^. Furthermore, these markers can be adapted for environmental DNA (eDNA) analyses, providing a powerful tool for species detection, particularly in difficult-to-survey habitats. Finally, these assembled mitogenomes enhance analytical accuracy by enriching reference databases^38^.

## Materials and methods

### Sample collection and DNA isolation

Two individuals, one male (Sample ID: CAT07) and one female (Sample ID: CAT08), were collected in accordance with the Hungarian nature conservation legislation (Act “1996. évi LIII. törvény a természet védelméről” 38. § (1) a) from one of the largest stable populations in Hungary near the settlement Vécs (location: N 47.7796° E 20.1532°), in late August 2020. The collection was conducted using an ultraviolet light trap and supervised by the zoological referee of the competent authority, the Bükk National Park Directorate. The sampling was carried out at the end of the species’ mating season. For the female specimen, the collected individual was also checked to verify if she had laid all her eggs prior to collection. The final two specimens selected for data generation were euthanized in accordance with the guidelines of the American Veterinary Medical Association (AVMA). Collected individuals were preserved in silica-gel and stored in a + 4 °C until DNA extraction to prevent DNA degradation. Genomic DNA was extracted from two legs of both individuals following the protocol of Bereczki et al. (2014)^39^. The DNA isolates were purified using AMPure XP magnetic beads (Beckman Coulter, Inc., Brea, CA, USA) following the manufacturer’s instructions. After the successful DNA extraction, the two specimens were preserved in the lepidopterological collection of the Department of Evolutionary Zoology, University of Debrecen (Hungary).

### Whole genome sequencing

A randomly sheared genomic library was prepared from 500 ng of genomic DNA using the MGIEasy Universal DNA Library Prep Set Kit v1.0 (MGI Tech Co., Ltd., Shenzhen, China), following the manufacturer’s instructions. The library was sequenced on an MGI DNBSEQ-G400RS instrument using the FCL PE150 sequencing set.

### Preprocessing of sequencing reads

To reduce the computational burden of *de novo* assembly, the reads were first aligned to the most closely related mitochondrial reference genome, *Eogystia hippophaecolus*^40^ (GenBank accession number NC_023936.1), the only member of the Cossinae subfamily with a published mitochondrial genome. Alignment was performed using BWA v07.17^41^, and then the aligned read pairs were identified using SAMtools v1.10^42^ for downstream analyses.

The quality of the mapped reads was assessed using fastqc v0.11.9^43^, then quality filtered using fastp v0.20.1^44^ with default settings. Due to the unbalanced base composition after approximately 100 base pairs (bp), the reads were trimmed to 100 bp, and sequences shorter than 95 bp were discarded using cutadapt v2.10^45^. Read error correction was performed using Bloocoo v1.0.6^46^.

### Assembly and analysis of the mitochondrial genome

The aligned reads were assembled using GetOrganelle v1.7.3.5^47^, with the animal mitochondrial genome specified as the target (-F animal_mt). The assembled mitogenomes were annotated using the MITOS2 server (http://mitos2.bioinf.uni-leipzig.de/index.py)^48^, and the integrated MiTFi^49^ performed the tRNA gene annotation and secondary structure prediction. Additionally, the mitochondrial genome assemblies were annotated by annotation transfer using Liftoff v1.6.3^50^ with the *Eogystia hippophaecolus* mitogenome annotation as a reference. For protein-coding genes (PCGs), if any differences were observed between the two annotations, the given sequences were carefully checked and aligned against the corresponding gene in the *E. hippophaecolus* to determine the most accurate annotation. The annotated mitogenome maps were visualized using the Proksee server (https://proksee.ca/)^51^.

Base composition, including percentage values for the individual nucleotides and AT% and GC%, was calculated according to the base counts of the genes and the control region. These values were further used to compute AT- and GC-skewness to describe the base composition of the assemblies. The skewness calculations followed the formulae of Perna & Kocher (1995)^52^: AT-skew=(A-T)/(A + T) and GC-skew=(G-C)/(G + C), where A, T, G, and C represent the counts of their respective nucleotides. Relative synonymous codon usage (RSCU) values for the mitochondrial protein-coding genes (PCGs) were calculated using Ezcodon^53^ implemented on the EZmito server (http://ezmito.unisi.it/ezcodon)^54^, by applying the invertebrate mitochondrial genetic code.

### Phylogenetic reconstruction

The phylogenetic relationships within the Cossoidea superfamily were inferred using the nucleotide sequences of the 13 mitochondrial PCGs (*atp6*, *atp8*, *cytb*, *cox1*, *cox2*, *cox3*, *nad1*, *nad2*, *nad3*, *nad4*, *nad4l*, *nad5*, *nad6*). Due to the limited number of sequenced mitochondrial genomes for the Cossoidea species, incomplete and unannotated sequences were included in the analysis. Two species from the Yponomeutoidea superfamily were included as outgroups (Table S1). For the annotated mitogenomes, nucleotide sequences of the PCGs were downloaded from GenBank. When annotations were unavailable, the mitogenome was annotated by Liftoff v1.6.347 with the *Eogystia hippophaecolus*mitogenome annotation serving as a reference. The PCGs were then extracted, separated by gene, and aligned with MACSE v2.06^55^, applying the invertebrate mitochondrial code (-gc_def 5). Then the aligned sequences were concatenated with AMAS^56^ resulting in an 11,250 bp alignment. Maximum Likelihood (ML) phylogenetic tree reconstruction was performed with IQ-TREE v2.0.3^57^. An edge-unlinked partition model that relied on ModelFinder Plus^58^ and the subsequential merging of genes (-m MFP + MERGE) was employed to identify the best-fitting evolutionary model for the dataset. Four runs were performed using different partitioning schemes: (I) merged sequences with all genes treated as distinct partitions (Fig. 2); (II) the first two codon positions and the third codon position defined as separate partitions (Fig. S4-A); (III) only the first two codon positions considered (Fig. S4-B); (IV) merged nucleotide sequences of the PCGs, using codon substitution models applying the invertebrate mitochondrial genetic code (Fig. S4-C). Statistical branch support was assessed using the SH-like approximate likelihood ratio test (SH-aLRT)^59^ and ultrafast bootstrap (UFBoot)^60^ with 10,000 replicates. Statistical support was considered to be existing only if SH-aLRT ≥ 80 and UFBoot ≥ 95.

## Electronic supplementary material

Below is the link to the electronic supplementary material.

Supplementary Material 1

## Data Availability

Raw reads used for the mitochondrial genome assembly were deposited in the SRA database under the BioProject PRJNA1231052, and the assembled mitogenomes were uploaded to the GenBank under accession numbers PQ668644 and PQ668645.

## References

[1] Minet, J. Tentative reconstruction of the Ditrysian phylogeny (Lepidoptera: Glossata). *Insect Syst. Evol.* **22**, 69–95 (1991).

[2] Regier, J. C. et al. A large-scale, higher-level, molecular phylogenetic study of the insect order Lepidoptera (moths and butterflies). *PloS ONE*. **8**, e58568 (2013). 23554903 10.1371/journal.pone.0058568 PMC3595289

[3] Heikkilä, M., Mutanen, M., Wahlberg, N., Sihvonen, P. & Kaila, L. Elusive Ditrysian phylogeny: an account of combining systematized morphology with molecular data (Lepidoptera). *BMC Evol. Biol.* **15**, 1–27 (2015). 26589618 10.1186/s12862-015-0520-0 PMC4654798

[4] Kawahara, A. Y. et al. Phylogenomics reveals the evolutionary timing and pattern of butterflies and moths. *Proc. Natl. Acad. Sci. USA*. **116**, 22657–22663 (2019). 31636187 10.1073/pnas.1907847116 PMC6842621

[5] Mayer, C. et al. Adding leaves to the Lepidoptera tree: capturing hundreds of nuclear genes from old museum specimens. *Syst. Entomol.* **46**, 649–671 (2021).

[6] Yang, X., Cameron, S. L., Lees, D. C., Xue, D. & Han, H. A mitochondrial genome phylogeny of Owlet moths (Lepidoptera: Noctuoidea), and examination of the utility of mitochondrial genomes for lepidopteran phylogenetics. *Mol. Phylogenet Evol.* **85**, 230–237 (2015). 25698356 10.1016/j.ympev.2015.02.005

[7] Kim, M. J., Wang, A. R., Park, J. S. & Kim, I. Complete mitochondrial genomes of five skippers (Lepidoptera: Hesperiidae) and phylogenetic reconstruction of Lepidoptera. *Gene* **549**, 97–112 (2014). 25058696 10.1016/j.gene.2014.07.052

[8] Timmermans, M. J., Lees, D. C. & Simonsen, T. J. Towards a mitogenomic phylogeny of Lepidoptera. *Mol. Phylogenet Evol.* **79**, 169–178 (2014). 24910155 10.1016/j.ympev.2014.05.031

[9] Chen, L. et al. Fourteen complete mitochondrial genomes of butterflies from the genus *Lethe* (Lepidoptera, nymphalidae, Satyrinae) with mitogenome-based phylogenetic analysis. *Genomics* **112**, 4435–4441 (2020). 32745503 10.1016/j.ygeno.2020.07.042

[10] Van Nieukerken, E. J. et al. Order Lepidoptera Linnaeus, 1758. In *Animal biodiversity: An outline of higher-level classification and survey of taxonomic richness. Zootaxa* , Vol. 3148 (ed Zhang, Z.-Q.) 212–221 (2011). 10.11646/zootaxa.3703.1.1 26146682

[11] Goldstein, P. Z. Diversity and significance of lepidoptera: A phylogenetic perspective. In *Insect biodiversity: Science and society* Vol. 1 (eds Foottit, R. G. & Adler, P. H.) 463–495 (Wiley, 2017).

[12] Mitter, C., Davis, D. R. & Cummings, M. P. Phylogeny and evolution of Lepidoptera. *Annu. Rev. Entomol.* **62**, 265–283 (2017). 27860521 10.1146/annurev-ento-031616-035125

[13] Sahoo, R. K. et al. Ten genes and two topologies: an exploration of higher relationships in skipper butterflies (Hesperiidae). *PeerJ* **4**, e2653 (2016). 27957386 10.7717/peerj.2653 PMC5144725

[14] Triant, D. A., Cinel, S. D. & Kawahara, A. Y. Lepidoptera genomes: current knowledge, gaps and future directions. *Curr. Opin. Insect Sci.* **25**, 99–105 (2018). 29602369 10.1016/j.cois.2017.12.004

[15] Schoorl, J. W. A phylogenetic study on Cossidae (Lepidoptera: Ditrysia) based on external adult morphology. *Zool. Verh*. **263**, 4–295 (1990).

[16] De Freina, J. J. Beitrag Zur systematischen erfassung der Bombyces-und Sphinges-fauna kleinasiens. Neue kenntnisse Uber artenspektrum, systematik und nomenklatur Sowie beschreibungen Neuer taxa (Lepidoptera). *Mitt Münch Entomol. Ges*. **72**, 57–127 (1983).

[17] Yakovlev, R. V. Catalogue of the family Cossidae of the old world. *Neue Ent Nachr.* **66**, 1–129 (2011).

[18] Hristova, H. & Beshkov, S. Checklist of the superfamilies cossoidea, thyridoidea, Drepanoidea, lasiocampoidea, Bombycoidea and noctuoidea: Notodontidae (Insecta: Lepidoptera) of Bulgaria, with application of the IUCN red list criteria at the National level. *Acta Zool. Bulg.* **68**, 569–576 (2016).

[19] Beshkov, S. & Nahirnić-Beshkova, A. *Paracossulus thrips* (Hübner, 1818) (Lep. Cossidae) rediscovered in Bulgaria with notes of some other surprising findings in the dragoman NATURA 2000 protected area. *Entomologist’s Rec J. Var.* **133**, 22–30 (2021).

[20] Beshkov, S. & Nahirnić-Beshkova, A. *Paracossulus thrips* (Hübner, 1818)(Cossidae) and *Lignyoptera fumidaria* (Hübner, 1825)(Geometridae)–two Lepidoptera genera new for Serbia with a review of the distribution of these two habitats directive species in the Balkan Peninsula. *Ecol. Montenegrina*. **51**, 65–80 (2022).

[21] Kemencei, Z. & Patalenszki, A. (eds) *Sztyeplepke [Steppe carpenter moth*]. in *Módszertani kézikönyv a hazánkban előforduló egyes közösségi jelentőségű állatfajok terepi vizsgálatához*. [*Methodological manual for the field survey of certain species of community importance in Hungary*] 237–245 (Ministry of Agriculture of Hungary, 2021).

[22] Iacob, G. M. et al. Improving the knowledge on distribution, food preferences and DNA barcoding of natura 2000 protected species *Paracossulus thrips* (Lepidoptera, Cossidae) in Romania. *Insects* **12**, 1087 (2021). 34940175 10.3390/insects12121087 PMC8703911

[23] Rákosy, L. et al. *Lista rosie a fluturilor din România. [Red List of Lepidoptera of Romania]* 67 (Presa Universitară Clujeană, 2021).

[24] Daniel, F. Monographie der palaearktischen Cossidae. V. Monographie der palaearktischen Cossidae. V. Die Genera *Parahypopta* gn, *Sinicossus* Clench und *Catopta* Stgr. *Mitt Münch Entomol. Ges* **51**, 160–212 (1961).

[25] Rakonczay, Z. (ed.) *Vörös könyv. A Magyarországon kipusztult és veszélyeztetett növény- és állatfajok.*[*Red book. Extinct and endangered plant and animal species in Hungary*] 189 (Akadémiai Kiadó, 1989).

[26] Sum, Sz. *Sztyepplepke Paracossulus thrips (Hübner, [1810–1813]. in Haraszthy, L. (Ed.) Natura 2000 fajok és élőhelyek Magyarországon.* [*Steppe carpenter moth Paracossulus thrips (Hübner, [1810–1813]. in Haraszthy, L. (Ed.) Natura 2000 species and sites in Hungary.*] 285–289 (Pro Vértes Közalapítvány, 2014).

[27] Liao, F. et al. The complete mitochondrial genome of the fall webworm, *Hyphantria cunea* (Lepidoptera: Arctiidae). *Int. J. Biol. Sci.* **6**, 172–186 (2010). 20376208 10.7150/ijbs.6.172 PMC2850540

[28] Chen, Q. et al. Comparative mitochondrial genome analysis and phylogenetic relationship among lepidopteran species. *Gene* **830**, 146516 (2022). 35452707 10.1016/j.gene.2022.146516

[29] Sun, Y. X. et al. Characterization of the complete mitochondrial genome of *Leucoma salicis* (Lepidoptera: Lymantriidae) and comparison with other lepidopteran insects. *Sci. Rep.* **6**, 39153 (2016). 27974854 10.1038/srep39153 PMC5156926

[30] Garey, J. R. & Wolstenholme, D. R. Platyhelminth mitochondrial DNA: evidence for early evolutionary origin of a tRNA Ser AGN that contains a dihydrouridine arm replacement loop, and of Serine-specifying AGA and AGG codons. *J. Mol. Evol.* **28**, 374–387 (1989). 2545889 10.1007/BF02603072

[31] Cameron, S. L. Insect mitochondrial genomics: implications for evolution and phylogeny. *Annu. Rev. Entomol.* **59**, 95–117 (2014). 24160435 10.1146/annurev-ento-011613-162007

[32] Zhao, M. Y., Huo, Q. B. & Du, Y. Z. Molecular phylogeny inferred from the mitochondrial genomes of Plecoptera with *Oyamia nigribasis* (Plecoptera: Perlidae). *Sci. Rep.* **10**, 20955 (2020). 33262442 10.1038/s41598-020-78082-y PMC7708463

[33] Saito, S., Tamura, K. & Aotsuka, T. Replication origin of mitochondrial DNA in insects. *Genetics* **171**, 1695–1705 (2005). 16118189 10.1534/genetics.105.046243 PMC1456096

[34] Cameron, S. L. & Whiting, M. F. The complete mitochondrial genome of the tobacco hornworm, *Manduca sexta*, (Insecta: lepidoptera: Sphingidae), and an examination of mitochondrial gene variability within butterflies and moths. *Gene* **408**, 112–123 (2008). 18065166 10.1016/j.gene.2007.10.023

[35] Blaxter, M. et al. Why sequence all eukaryotes? *Proc. Natl. Acad. Sci. USA*. **119**, e2115636118 (2022). 35042801 10.1073/pnas.2115636118 PMC8795522

[36] Sittenthaler, M. et al. DNA barcoding of exuviae for species identification of central European damselflies and dragonflies (Insecta: Odonata). *J. Insect Conserv.* **27**, 435–450 (2023).

[37] Li, C. et al. Mitochondrial genome provides species-specific targets for the rapid detection of early invasive populations of Hylurgus ligniperda in China. *BMC Genom.* **25**, 90 (2024). 10.1186/s12864-024-10011-z PMC10804472 38254044

[38] Chua, P. Y. et al. Future of DNA-based insect monitoring. *Trends Genet.* **39**, 531–544 (2023). 36907721 10.1016/j.tig.2023.02.012

[39] Bereczki, J., Tóth, J. P., Sramkó, G. & Varga, Z. Multilevel studieson the two phenological forms of large blue (*Maculinea arion*)(Lepidoptera: Lycaenidae). *J. Zoolog Syst. Evol. Res.* **52**, 32–43 (2014).

[40] Gong, Y. J., Wu, Q. L. & Wei, S. J. The first complete mitogenome for the superfamily Cossoidea of lepidoptera: the Seabuckthorn carpenter moth *Eogystia Hippophaecolus*. *Mitochondrial DNA*. **25**, 288–289 (2014). 23841606 10.3109/19401736.2013.792071

[41] Li, H. & Durbin, R. Fast and accurate short read alignment with Burrows-Wheeler transform. *Bioinformatics* **25**, 1754–1760 (2009). 19451168 10.1093/bioinformatics/btp324 PMC2705234

[42] Li, H. et al. The sequence alignment/map format and samtools. *Bioinformatics* **25**, 2078–2079 (2009). 19505943 10.1093/bioinformatics/btp352 PMC2723002

[43] Andrews, S. FastQC: a quality control tool for high throughput sequence data. https://www.bioinformatics.babraham.ac.uk/projects/fastqc/ (Babraham Bioinformatics, Babraham Institute, Cambridge, United Kingdom, 2010).

[44] Chen, S., Zhou, Y., Chen, Y. & Gu, J. Fastp: an ultra-fast all-in-one FASTQ preprocessor. *Bioinformatics* **34**, i884–i890 (2018). 30423086 10.1093/bioinformatics/bty560 PMC6129281

[45] Martin, M. Cutadapt removes adapter sequences from high-throughput sequencing reads. *EMBnet J.* **17**, 10–12 (2011).

[46] Benoit, G., Lavenier, D., Lemaitre, C., & Rizk, G. Bloocoo, a memory efficient read corrector. in *European conference on computational biology (ECCB)* (2014).

[47] Jin, J. J. et al. GetOrganelle: a fast and versatile toolkit for accurate de Novo assembly of organelle genomes. *Genome Biol.* **21**, 1–31 (2020). 10.1186/s13059-020-02154-5 PMC7488116 32912315

[48] Donath, A. et al. Improved annotation of protein-coding genes boundaries in metazoan mitochondrial genomes. *Nucleic Acids Res.* **47**, 10543–10552 (2019). 31584075 10.1093/nar/gkz833 PMC6847864

[49] Jühling, F. et al. Improved systematic tRNA gene annotation allows new insights into the evolution of mitochondrial tRNA structures and into the mechanisms of mitochondrial genome rearrangements. *Nucleic Acids Res.* **40**, 2833–2845 (2012). 22139921 10.1093/nar/gkr1131 PMC3326299

[50] Shumate, A., Salzberg, S. L. & Liftoff Accurate mapping of gene annotations. *Bioinformatics* **37**, 1639–1643 (2021). 33320174 10.1093/bioinformatics/btaa1016 PMC8289374

[51] Grant, J. R. et al. Proksee: in-depth characterization and visualization of bacterial genomes. *Nucleic Acids Res.* **51**, W484–W492. 10.1093/nar/gkad326 (2023). 37140037 10.1093/nar/gkad326 PMC10320063

[52] Perna, N. T. & Kocher, T. D. Patterns of nucleotide composition at fourfold degenerate sites of animal mitochondrial genomes. *J. Mol. Evol.* **41**, 353–358 (1995). 7563121 10.1007/BF00186547

[53] Lee, B. D. Python implementation of codon adaptation index. *J. Open. Source Softw.* **3**, 905. 10.21105/joss.00905 (2018).

[54] Cucini, C. et al. EZmito: a simple and fast tool for multiple mitogenome analyses. *Mitochondrial DNA B Resour.* **6**, 1101–1109 (2021). 33796755 10.1080/23802359.2021.1899865 PMC7995877

[55] Ranwez, V., Douzery, E. J., Cambon, C., Chantret, N. & Delsuc, F. MACSE v2: toolkit for the alignment of coding sequences accounting for frameshifts and stop codons. *Mol. Biol. Evol.* **35**, 2582–2584 (2018). 30165589 10.1093/molbev/msy159 PMC6188553

[56] Borowiec, M. L. AMAS: a fast tool for alignment manipulation and computing of summary statistics. *PeerJ* **4**, e1660 (2016). 26835189 10.7717/peerj.1660 PMC4734057

[57] Nguyen, L. T., Schmidt, H. A., Von Haeseler, A. & Minh, B. Q. IQ-TREE: A fast and effective stochastic algorithm for estimating Maximum-Likelihood phylogenies. *Mol. Biol. Evol.* **32**, 268–274 (2015). 25371430 10.1093/molbev/msu300 PMC4271533

[58] Kalyaanamoorthy, S., Minh, B. Q., Wong, T. K., Von Haeseler, A. & Jermiin, L. S. ModelFinder: fast model selection for accurate phylogenetic estimates. *Nat. Methods*. **14**, 587–589 (2017). 28481363 10.1038/nmeth.4285 PMC5453245

[59] Guindon, S. et al. New algorithms and methods to estimate maximum-likelihood phylogenies: assessing the performance of PhyML 3.0. *Syst. Biol.* **59**, 307–321 (2010). 20525638 10.1093/sysbio/syq010

[60] Minh, B. Q. & Nguyen, M. A. T. Von Haeseler, A. Ultrafast approximation for phylogenetic bootstrap. *Mol. Biol. Evol.* **30**, 1188–1195 (2013). 23418397 10.1093/molbev/mst024 PMC3670741

