# Supplementary material for: The mitochondrial genome of the steppe carpenter moth (Paracossulus thrips Hübner, 1818): Structural analysis and phylogenetic implications
Source: Sci Rep. 2025 Mar 11;15:8393. doi: 10.1038/s41598-025-93646-6 (PMC11897335; doi:10.1038/s41598-025-93646-6)
Supplement: Supplementary file 1 — Supplementary Material 1 [file 41598_2025_93646_MOESM1_ESM.docx]

**Supplementary Material for Jordán et al.: The mitochondrial genome of the Steppe carpenter moth (*Paracossulus thrips* Hübner, 1818)—structural analysis and phylogenetic implications**

**Supplementary Tables**

**Table S1** Summary of samples involved in phylogenetic reconstruction.

| **NCBI accession number** | **Length (bp)** | **Species** | **Superfamily** | **Family** | **Subfamily** |
| --- | --- | --- | --- | --- | --- |
| NC_037944.1 | 15,259 | *Lyonetia clerkella* | Yponomeutoidea | Lyonetiidae | - |
| NC_025322.1 | 16,014 | *Plutella xylostella* | Yponomeutoidea | Plutellidae | - |
| NC_023936.1 | 15,431 | *Eogystia hippophaecolus* | Cossoidea | Cossidae | Cossinae |
| NC_051865.1 | 15,490 | *Chalcidica minea* | Cossoidea | Cossidae | Zeuzerinae |
| NC_062621.1 | 15,285 | *Endoxyla cinereus* | Cossoidea | Cossidae | Zeuzerinae |
| OZ195577.1 | 15,496 | *Phragmataecia castaneae* | Cossoidea | Cossidae | Zeuzerinae |
| KJ508046.1 | 7,983 | *Zeuzera coffeae* | Cossoidea | Cossidae | Zeuzerinae |
| NC_051866.1 | 15,320 | *Zeuzera multistrigata* | Cossoidea | Cossidae | Zeuzerinae |
| MF491642.1 | 15,260 | *Zeuzera multistrigata* | Cossoidea | Cossidae | Zeuzerinae |
| OP379744.1 | 15,324 | *Zeuzera pyrina* | Cossoidea | Cossidae | Zeuzerinae |
| OU015648.1 | 15,281 | *Zeuzera pyrina* | Cossoidea | Cossidae | Zeuzerinae |

**Table S2** Summary of mutations and variable sites found between the assembled *Paracossulus thrips* mitochondrial genomes.

| **Position** | **Region** | **Mutation** | | **Type** | **Codon** |
| --- | --- | --- | --- | --- | --- |
|  |  | **CAT07** | **CAT08** |  |  |
| 16 | tRNA-*Met* (CAU) | G | A | transition | – |
| 1,408–1,413 | tRNA-Cys (GCA)  –  tRNA-*Tyr* (GUA) | – | (AT)_3_ | microsatellite | – |
| 1,430-1,433 | tRNA-*Cys* (GCA)  –  tRNA-*Tyr* (GUA) | AAAT | – | microsatellite | – |
| 2,075 | *cox1* | G | A | transition | GCC→ACC  (Ala→Thr) |
| 4,996 | *cox3* | A | G | transition | AGA→GGA  (Ser→Gly) |
| 6,359–6,368 | tRNA-*Glu* (UUC)  –  tRNA-*Phe* (GAA) | (TA)_5_ | – | microsatellite | – |
| 9,779 | *nad4l* | T | A | transversion | ATA→ATT  (Met→Ile) |
| 11,940 | *nad1* | G | A | transition | CCT→TCT  (Pro→Ser) |
| 13,083 | 16S rRNA | A | – | indel | – |
| 13,629 | 16S rRNA | T | – | indel | – |
| 15,270 | CR | G | A | transition | – |

In the „Codon” column, the amino acid abbreviations in brackets following the codon sequences indicate the amino acid substitutions caused by non-synonymous mutations identified in the respective codons.

**Table S3** Base composition of the mitogenome assemblies.

| **Region** | **A (%)** | **C (%)** | **G (%)** | **T (%)** | **A+T (%)** | **G+C (%)** | **AT–skewness** | **GC–skewness** |
| --- | --- | --- | --- | --- | --- | --- | --- | --- |
| Mitogenome | 39.9 | 14.5 | 7.8 | 37.8/37.7 | 77.7 | 22.3 | 0.03 | -0.30 |
| *atp6* | 33.9 | 16.7 | 8.7 | 40.7 | 74.6 | 25.4 | -0.09 | -0.31 |
| *atp8* | 43.6 | 9.7 | 1.8 | 44.8 | 88.5 | 11.5 | -0.01 | -0.68 |
| *cob* | 32.5 | 18.9 | 10.4 | 38.2 | 70.7 | 29.3 | -0.08 | -0.29 |
| *cox1* | 30.6/30.7 | 18.1 | 13.8/13.7 | 37.5 | 68.1/68.2 | 31.9/31.8 | -0.10 | -0.13/-0.14 |
| *cox2* | 35.6 | 16.4 | 9.5 | 38.4 | 74.0 | 26.0 | -0.04 | -0.27 |
| *cox3* | 32.4/32.3 | 18.0 | 12.4/12.5 | 37.1 | 69.6/69.5 | 30.4/30.5 | -0.07 | -0.18 |
| *nad1* | 26.9 | 7.5/7.4 | 17.4 | 48.2/48.3 | 75.1/75.2 | 24.9/24.8 | -0.28/-0.29 | 0.40 |
| *nad2* | 34.5 | 13.4 | 6.4 | 45.7 | 80.2 | 19.8 | -0.14 | -0.35 |
| *nad3* | 36.4 | 15.3 | 6.8 | 41.5 | 78.0 | 22.0 | -0.07 | -0.38 |
| *nad4* | 31.6 | 7.5 | 14.5 | 46.4 | 78.0 | 22.0 | -0.19 | 0.32 |
| *nad4l* | 29.2/28.8 | 4.9 | 15.6 | 50.3/50.7 | 79.5 | 20.5 | -0.27/-0.28 | 0.53 |
| *nad5* | 30.3 | 6.2 | 15.1 | 48.3 | 78.6 | 21.4 | -0.23 | 0.42 |
| *nad6* | 35.2 | 11.9 | 6.4 | 46.5 | 81.7 | 18.3 | -0.14 | -0.30 |
| 12S rRNA | 44.1 | 4.8 | 10.1 | 41.1 | 85.2 | 14.8 | 0.03 | 0.36 |
| 16S rRNA | 39.2 | 5.0 | 12.9/13 | 42.8 | 82.0 | 18.0 | -0.04 | 0.44 |
| tRNA-*Ala* (UGC) | 41.2 | 7.4 | 7.4 | 44.1 | 85.3 | 14.7 | -0.03 | 0.00 |
| tRNA-*Arg* (UCG) | 39.1 | 12.5 | 12.5 | 35.9 | 75.0 | 25.0 | 0.04 | 0.00 |
| tRNA-*Asn* (GUU) | 43.1 | 9.2 | 10.8 | 36.9 | 80.0 | 20.0 | 0.08 | 0.08 |
| tRNA-*Asp* (GUC) | 46.3 | 9.0 | 4.5 | 40.3 | 86.6 | 13.4 | 0.07 | -0.33 |
| tRNA-*Cys* (GCA) | 39.1 | 5.8 | 11.6 | 43.5 | 82.6 | 17.4 | -0.05 | 0.33 |
| tRNA-*Gln* (UUG) | 37.7 | 4.3 | 11.6 | 46.4 | 84.1 | 15.9 | -0.10 | 0.45 |
| tRNA-*Glu* (UUC) | 49.3 | 4.3 | 2.9 | 43.5 | 92.8 | 7.2 | 0.06 | -0.20 |
| tRNA-*Gly* (UCC) | 43.3 | 4.5 | 4.5 | 47.8 | 91.0 | 9.0 | -0.05 | 0.00 |
| tRNA-*His* (GUG) | 43.3 | 4.5 | 9.0 | 43.3 | 86.6 | 13.4 | 0.00 | 0.33 |
| tRNA-*Ile* (GAU) | 35.9 | 9.4 | 15.6 | 39.1 | 75.0 | 25.0 | -0.04 | 0.25 |
| tRNA-*Leu* (UAA) | 37.1 | 12.9 | 12.9 | 37.1 | 74.3 | 25.7 | 0.00 | 0.00 |
| tRNA-*Leu* (UAG) | 43.5 | 4.3 | 13.0 | 39.1 | 82.6 | 17.4 | 0.05 | 0.50 |
| tRNA-*Lys* (UUU) | 38.9 | 13.9 | 12.5 | 34.7 | 73.6 | 26.4 | 0.06 | -0.05 |
| tRNA-*Met* (CAU) | 39.4/40.9 | 13.6 | 13.6/12.1 | 33.3 | 72.7/74.2 | 27.3/25.8 | 0.08/0.10 | 0/-0.06 |
| tRNA-*Phe* (GAA) | 40.3 | 4.5 | 13.4 | 41.8 | 82.1 | 17.9 | -0.02 | 0.50 |
| tRNA-*Pro* (UGG) | 40.9 | 6.1 | 15.2 | 37.9 | 78.8 | 21.2 | 0.04 | 0.43 |
| tRNA-*Ser* (UCU) | 40.9 | 9.1 | 9.1 | 40.9 | 81.8 | 18.2 | 0.00 | 0.00 |
| tRNA-*Ser* (UGA) | 40.3 | 6.0 | 11.9 | 41.8 | 82.1 | 17.9 | -0.02 | 0.33 |
| tRNA-*Thr* (UGU) | 40.0 | 7.7 | 9.2 | 43.1 | 83.1 | 16.9 | -0.04 | 0.09 |
| tRNA-*Trp* (UCA) | 44.3 | 11.4 | 7.1 | 37.1 | 81.4 | 18.6 | 0.09 | -0.23 |
| tRNA-*Tyr* (GUA) | 36.9 | 7.7 | 18.5 | 36.9 | 73.8 | 26.2 | 0.00 | 0.41 |
| tRNA-*Val* (UAC) | 44.9 | 7.2 | 14.5 | 33.3 | 78.3 | 21.7 | 0.15 | 0.33 |
| CR | 43.7/44.0 | 4.5 | 2.1/1.9 | 49.6 | 93.3/93.6 | 6.7/6.4 | -0.06 | -0.36/-0.42 |

Values separated by a slash (“/”) indicate differences in the counts of samples within a given region, with the values corresponding to CAT07 and CAT08 respectively.

**Supplementary Figures**

**
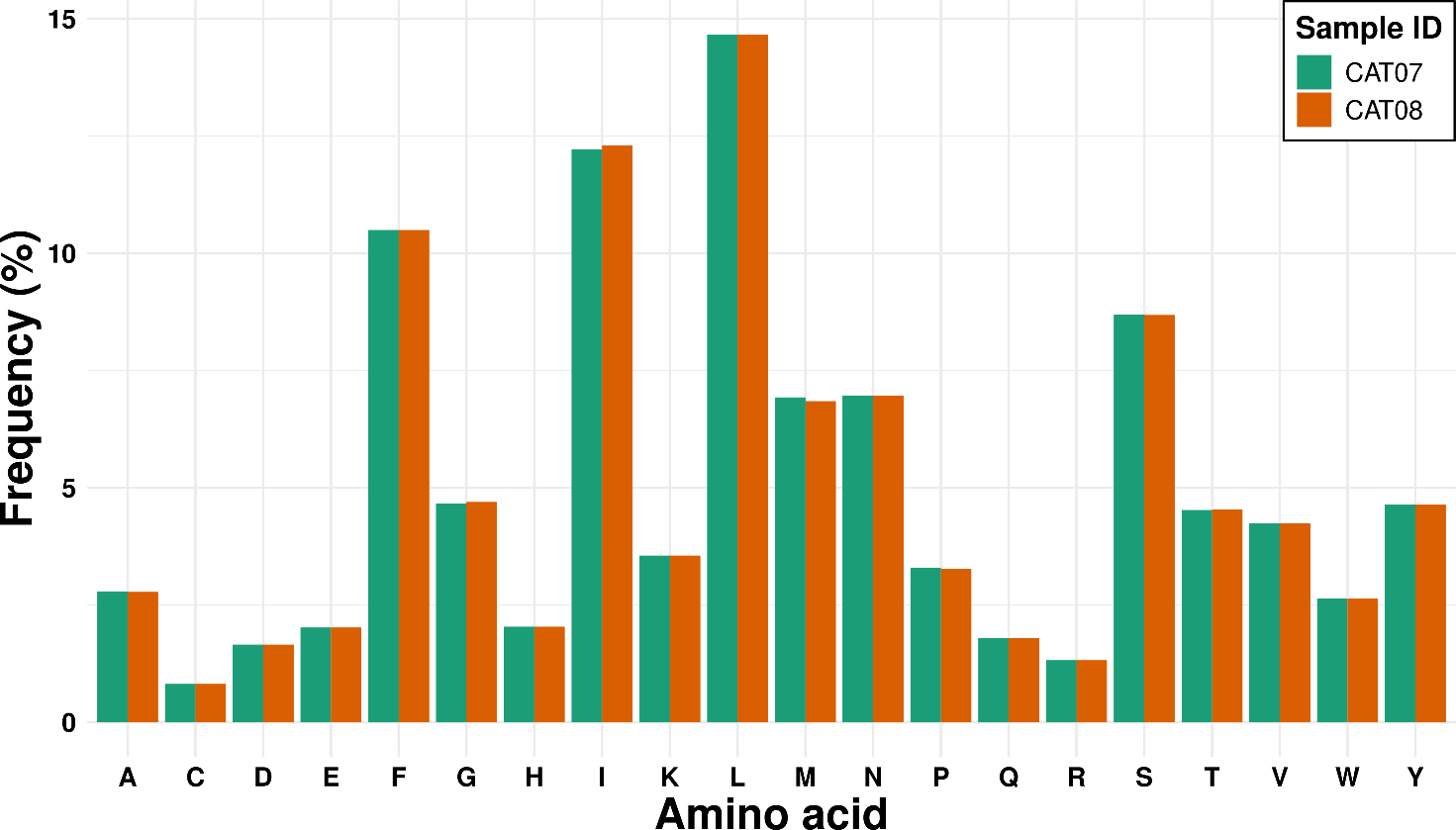
Fig. S1** Observed amino acid frequency in the PCGs of the assembled mitochondrial genomes. The letters on the x-axis represent the IUPAC amino acid codes for each amino acid.

**
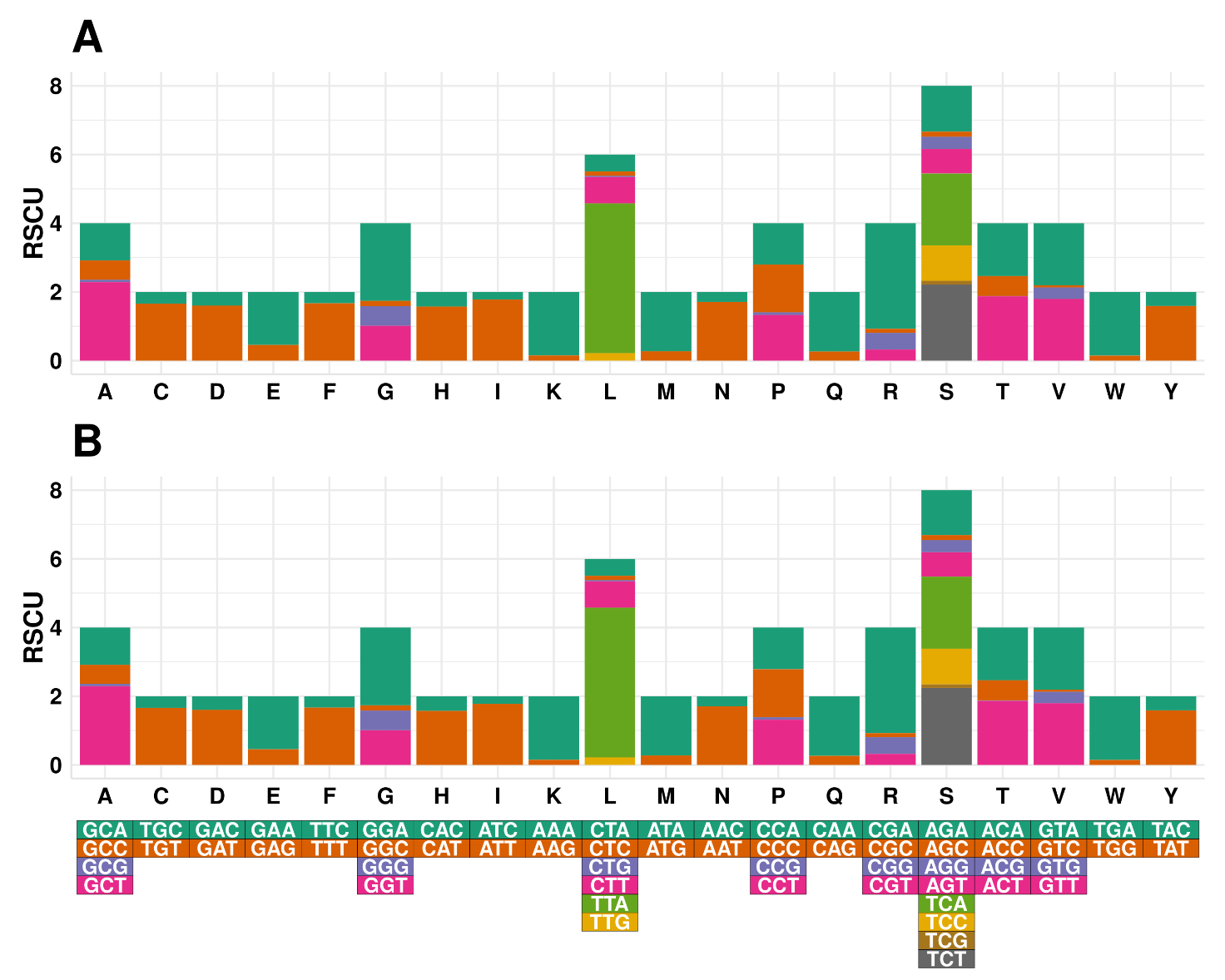
Fig. S2** Relative synonymous codon usage (RSCU) of the PCGs of the assembled mitochondrial genomes. **A**) RSCU plot of CAT07; **B**) RSCU plot of CAT08. The letters on the x-axis correspond to the IUPAC amino acid code of the given amino acids.

**
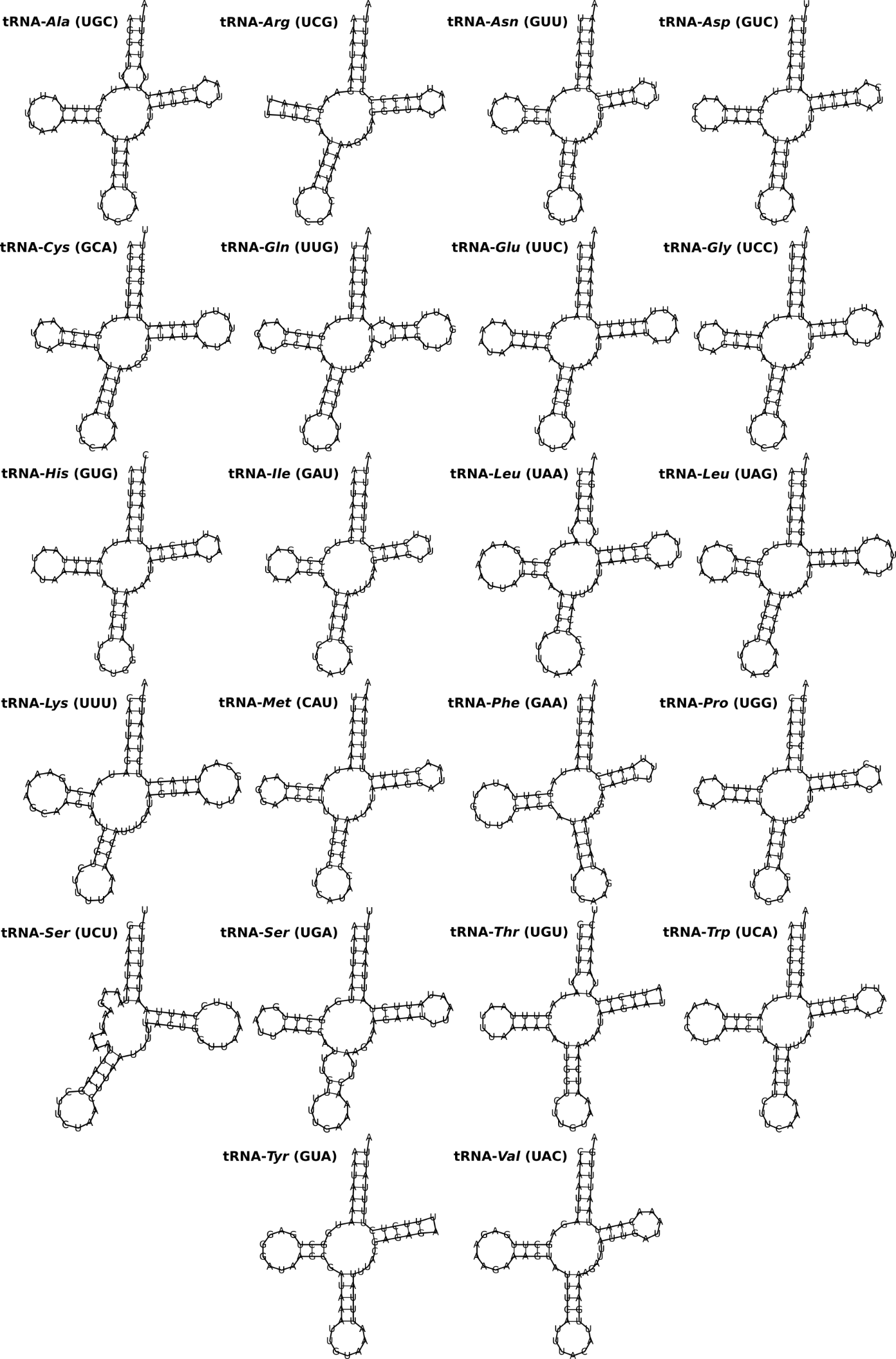
Fig. S3** Predicted secondary structures of the tRNA genes identified in the assembled mitochondrial genomes of *Paracossulus thrips*.

**
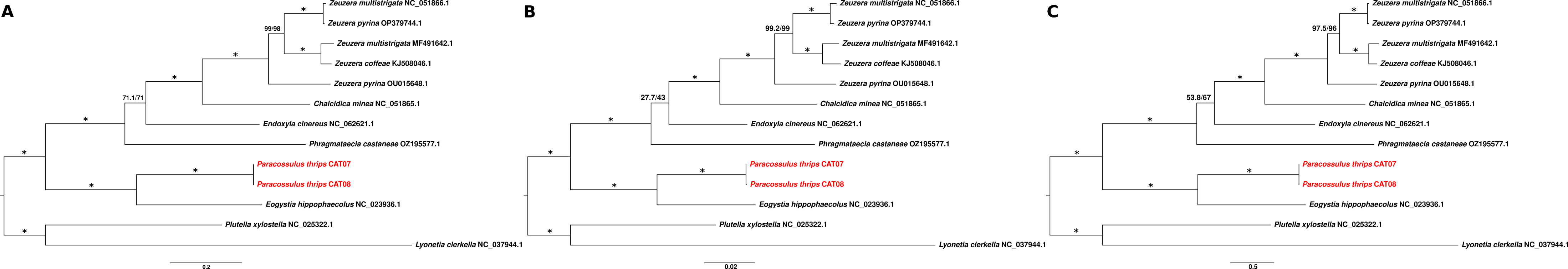
**  **
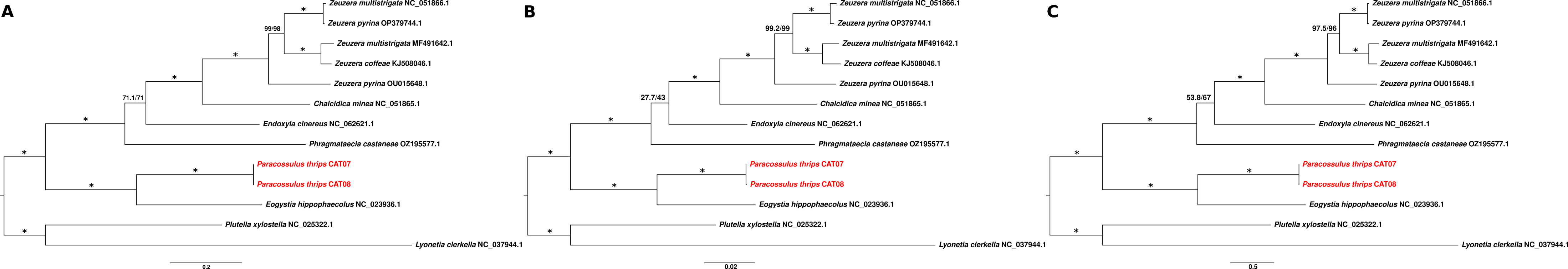

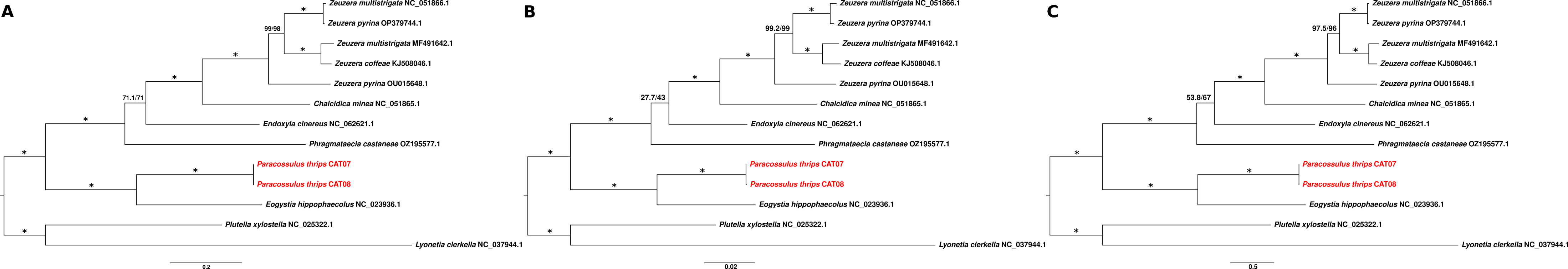
**

**Fig. S4** ML phylogenetic trees of Cossidae using 13 mitochondrial PCGs from the available mitogenomes using different partition schemes: **A**) merged set of the nucleotide sequences, with the first two and the third codon positions treated as distinct partitions for each gene; **B**) reconstruction based on the first and the second codon positions of the genes; **C**) reconstruction using codon substitution models, and the invertebrate mitochondrial genetic code. Branch support values representing SH-like approximate likelihood ratio test (SH-aLRT) and ultrafast bootstrap (UFBoot) with 10,000 replicates. Branches with 100/100 statistical support are indicated by an asterisk (*).
